# Nanopore sequencing in infectious disease diagnostics and surveillance

**DOI:** 10.1128/jcm.00083-26

**Published:** 2026-09-17

**Authors:** Adithi Somayaji, Piyas Mukherjee, Caroline Koch, Clarissa Prazeres da Costa, Youssef Hamway

**Affiliations:** 1 Institute for Medical Microbiology, Immunology and Hygiene, TUM School of Medicine and Health, Technical University of Munich (TUM), Munich, Germany; 2 Center for Global Health, TUM School of Medicine and Health, Technical University of Munich (TUM) 9184, Munich, Germany; 3 German Center for Infection Research (DZIF), partner site Munich 655317, Munich, Germany; 4 Department of Chemistry, Molecular Science Research Hub, Imperial College London, White City Campus 218235, London, United Kingdom; 5 Department of Life Sciences, Sir Alexander Fleming Building, Imperial College London, South Kensington Campus 98455, London, United Kingdom; Medical College of Wisconsin, Milwaukee, Wisconsin, USA

**Keywords:** infectious diseases, diagnostics, surveillance, AMR, emerging pathogens, nanopore sequencing

## Abstract

The global burden of infectious disease is on the rise, driven by antibiotic resistance, emerging pathogens, and gaps in accessible, decentralized diagnostics. To address these demands, there is a need for the development of diagnostic platforms for detection, surveillance, or monitoring assays with quick turnaround times. Oxford Nanopore sequencing, a platform for real-time long-read third-generation sequencing, has emerged as a promising tool in this space, with the ability to detect a wide range of molecular targets, including DNA, RNA, proteins, and base modifications. Its unique advantages, including portability, scalability, and the capacity for culture-independent workflows, combined with the depth of sequencing data produced, make it especially well-suited for infectious disease diagnostics and surveillance in both clinical and field settings. In this review, we aim to critically examine recent advances in the application of nanopore sequencing for diagnosing infectious diseases caused by bacterial, viral, fungal, and parasitic agents, as well as for detecting antimicrobial resistance genes. We also compare its potential advantages over conventional diagnostic methods and explore its expanding role in tracking outbreaks, monitoring pathogens in the environment, and detecting spillover events from animals to humans. Together, these applications highlight the relevance and position of this novel technology as a tool of choice for translational medical research and clinicians in the fight against infectious diseases and for global health preparedness.

## INTRODUCTION

Infectious diseases represent an escalating global health burden, contributing substantially to mortality and morbidity worldwide (1). These infections are driven by diverse biological agents, including bacteria (415 million DALYs; 58.9%), viruses and prions (178 million DALYs; 25.3%), parasites (72 million DALYs; 10.2%), and fungal and other emerging pathogens (2, 3). Importantly, a substantial proportion of this burden is preventable through early diagnosis and robust surveillance enabling timely treatment and interruption of transmission.

Additionally, infectious disease burdens remain disproportionately concentrated in low- and lower-middle-income countries (LMICs), particularly in South-East Asia and Africa, and are further amplified by anthropogenic factors (behavioral, occupational, metabolic) and environmental pressures, such as climate change (3). Together, these trends present a growing challenge for global health systems. Limited access to decentralized diagnostic tools further exacerbates the global burden of infectious diseases by hindering rapid detection and control. Additionally, strengthening disease surveillance systems can help reduce the global burden of infectious diseases by enabling earlier detection and response to emerging infections and zoonotic spillover events.

Accordingly, The Lancet One Health Commission suggests that robust surveillance is best achieved through a One Health perspective that recognizes the interconnections between human, animal, and environmental health (4). Recommending frameworks that support interoperable data sharing to integrate human, animal, and environmental pathogen data, strengthen equitable access to sequencing and capacity building in LMICs, and embed genomic surveillance within community-centered, multisectoral systems that link detection directly to public health action. Therefore, there is a growing impetus on researchers to pursue novel technologies and platforms for diagnostics and surveillance development, including sequencing approaches.

Genomic sequencing, once limited to room-sized laboratories, is now possible with increasingly pocket-sized devices (5), offering promise for closing the diagnostic gap in infectious diseases through the democratization of sequencing platforms. This evolution is driven in part by companies offering third-generation sequencing technology that promises long reads and real-time data generation. One such technology developed by Oxford Nanopore Technologies (ONT) is discussed in this review. Nanopore sequencing offers key advantages, including portability (MinION specifically is portable and USB-connected), relatively low initial cost of the device compared to other sequencers, and scalability, making it valuable for on-site diagnostics and surveillance, particularly in resource-limited settings (6). However, several challenges remain, including dependence on cold-chain storage for reagents, substantial computational requirements (including GPU support), downstream bioinformatics analyses, and the need for specialized training and expertise. Additionally, the cost of ONT platforms and consumables remains higher compared to current gold-standard molecular diagnostic methods, such as PCR.

Technically, ONT sequencing works by threading individual DNA or RNA molecules through protein nanopores embedded in a synthetic membrane. An applied electric field drives ionic current through each pore, and the characteristic disruptions caused by each passing nucleotide are decoded in real time by a basecalling algorithm (7). This architecture, distinct from sequencing-by-synthesis approaches, uniquely enables direct RNA sequencing and detection of base modifications without amplification. Novel approaches like nanopore-based sensing of DNA barcoded probes to enable detection of soluble biomarkers like microRNAs, proteins, etc. (8), expand the range of molecular markers that can be targeted.

This review critically evaluates the current role of nanopore sequencing, particularly ONT, in infectious disease diagnosis and surveillance across clinical, public health, and One Health contexts. We examine the potential of nanopore sequencing, including its applications in diagnostics, antimicrobial resistance detection, outbreak tracking, and environmental pathogen monitoring.

## APPLICATION OF NANOPORE SEQUENCING IN DIAGNOSTICS

Existing diagnostic tools, including culture, microscopy, serology, and targeted PCR, each carry inherent trade-offs in turnaround time, sensitivity, breadth of detection, or infrastructure requirements that limit their utility in complex clinical cases and LMICs. For fastidious, slow-growing, or polymicrobial infections in particular, current methods frequently fail to provide timely or comprehensive answers. Nanopore sequencing has emerged as a complementary platform capable of addressing several of these gaps, offering culture-independent, broad-spectrum pathogen detection with real-time data generation, and antimicrobial resistance identification.

A critical consideration throughout this review is that “nanopore sequencing” does not refer to a single, unified diagnostic assay. Rather, ONT sequencing encompasses a family of workflows that share the same analytic sequencing step but differ markedly in every other component, and these differences have a substantial and demonstrated impact on diagnostic performance.

**Fig 1:**
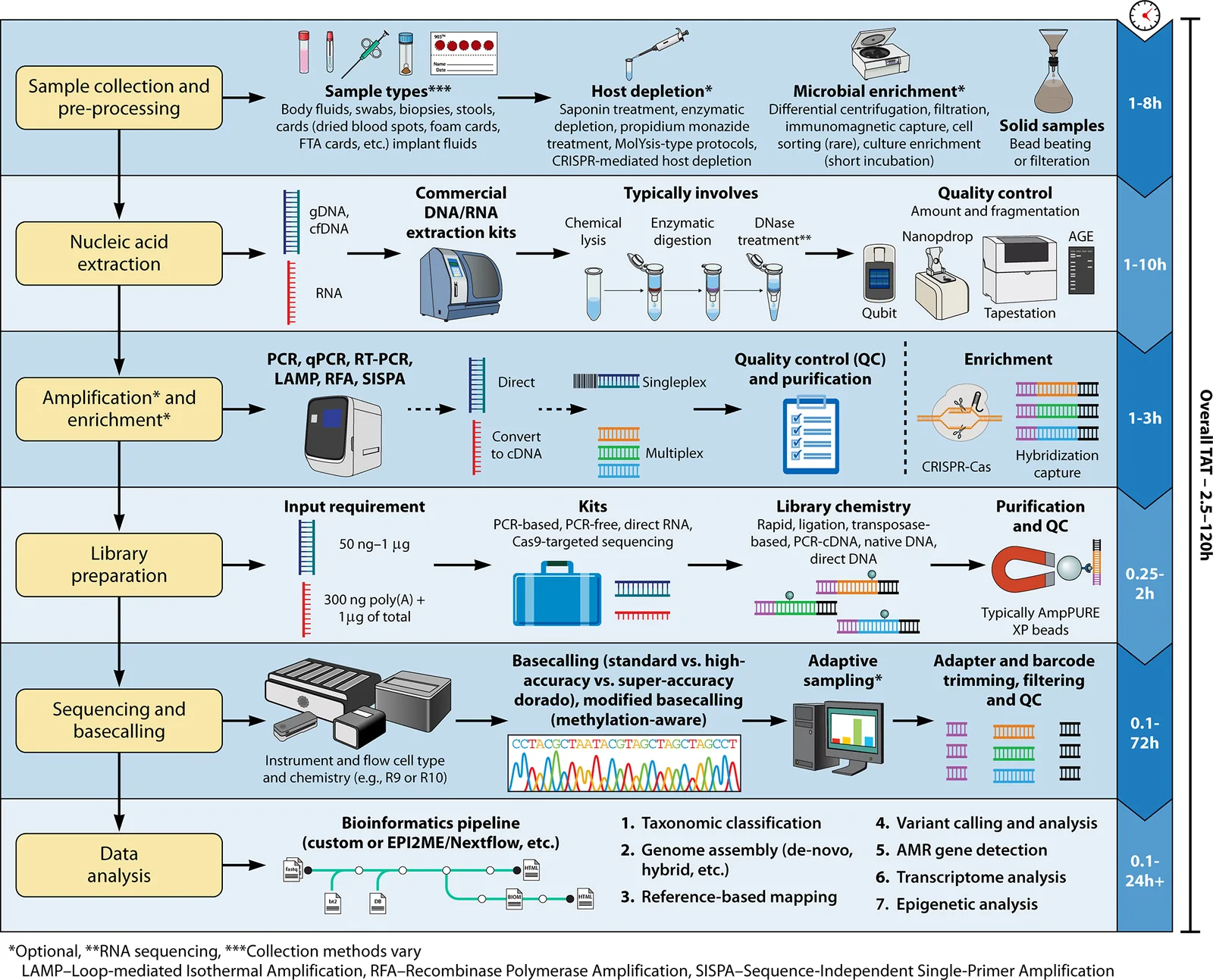
Overview of typical steps used for nanopore sequencing workflow for infectious disease diagnostics and surveillance in the cited studies. A schematic overview summarizes the key steps commonly involved in nanopore sequencing workflows, including sample processing, nucleic acid extraction, amplification, library preparation, sequencing, base calling, and data analysis. The exact workflow and order of steps may vary depending on the specific sequencing approach used. Key methods, approximate time estimates, and quality control steps are highlighted across different workflow types described in the cited in this review.

As described in Fig. 1, at the pre-analytical stage, workflows vary in: specimen type (sterile vs non-sterile; liquid vs solid); host-depletion strategy (saponin lysis, enzymatic depletion, adaptive sampling, propidium monoazide treatment); nucleic acid extraction method; amplification approach (PCR-based, isothermal, unamplified); enrichment strategy (CRISPR-Cas, hybridization capture, adaptive sequencing); and library preparation chemistry (rapid, ligation, transposase-based) (9).

At the analytic stage, key variables include flow cell chemistry (e.g., R9.4.1 vs R10.4.1, which differ in homopolymer accuracy and overall read quality), instrument platform (MinION, GridION, or PromethION, with varying throughput and field-deployability profiles), run duration, and multiplexing approach. Post-analytically, outcomes are shaped by the basecalling model used (e.g., standard vs high-accuracy vs super-accuracy Dorado, with differing GPU requirements), QC thresholds applied, choice of taxonomic classifier and reference database (e.g., Kraken2/RefSeq vs Minimap2/custom vs Kaiju), AMR gene database (e.g., CARD, ResFinder, and NCBI AMRFinder), bioinformatic pipeline (in-house vs standard; e.g., EPI2ME/Nextflow, etc.) and clinical reporting thresholds (9).

**Fig 2:**
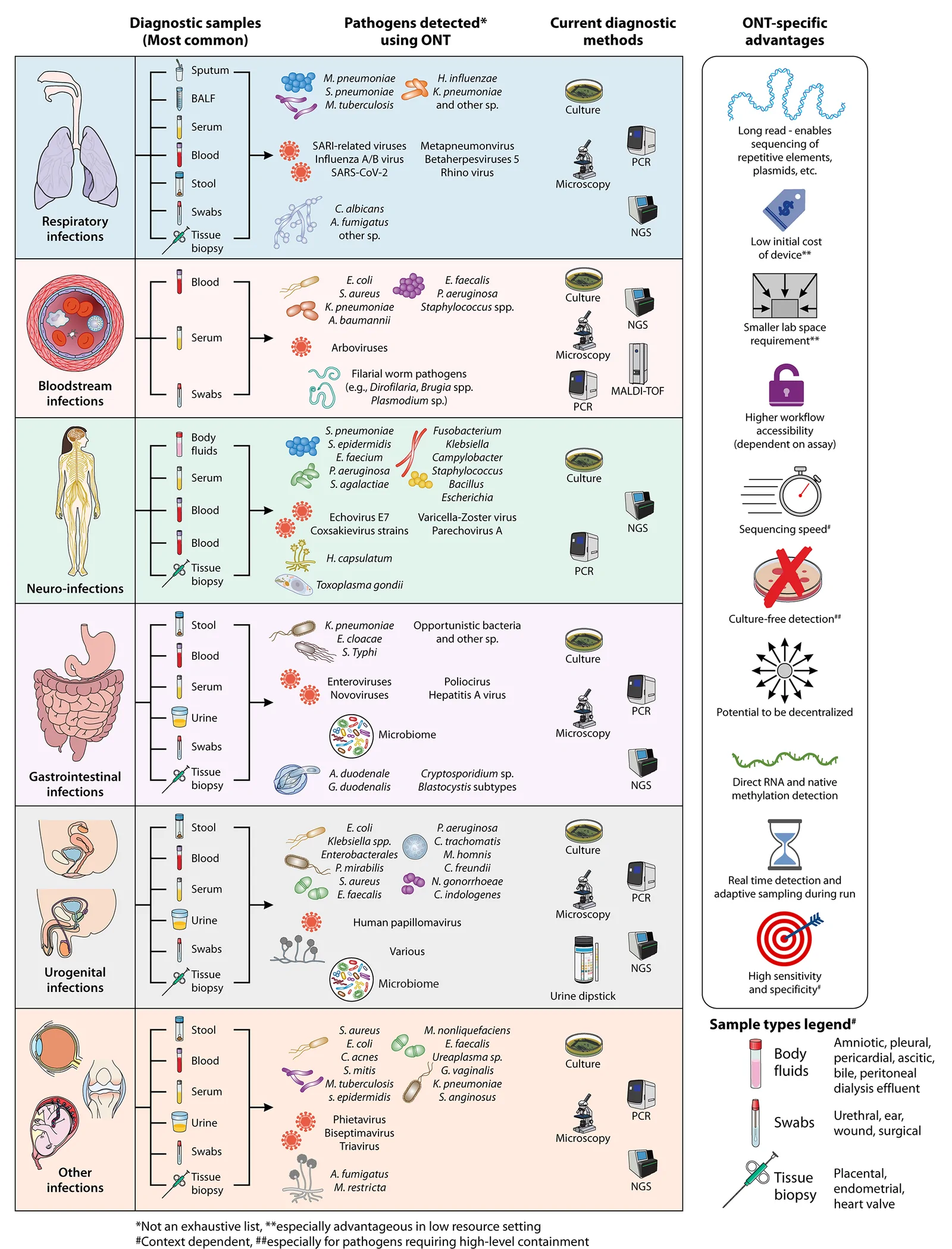
Diagnostic modalities in the context of organ or site of infection and their targeting pathogens, as employed in different infectious diseases. A graphical summary of diagnostic modalities is shown, varying across target organs for pathogen infection and sample collection in humans and currently employed diagnostic methods in different infectious disease settings. Divided by organ or associated systems (lung and respiratory tract; blood circulatory system; brain and CNS; gastrointestinal tract; kidney and renal system; connective tissue samples from joints, ocular chamber, or amniotic fluids) infected with their respective source of infection (including bacterial, fungal, viral, or parasitic agents). More information has been provided in Table S1.

This inherent flexibility in workflow design is also what enables nanopore sequencing to be applied across multiple infection systems in research contexts, including respiratory (10, 11), gastrointestinal (12), urogenital (13–16), neurological (17), bone/joint (18), bloodstream (19), and ocular (20, 21) or intra-amniotic infections (22). As shown in Fig. 2, consequently, it has been validated across a wide range of clinical specimens, like body fluids, such as urine (15, 16) and blood (19), tissue biopsies (placental, endometrial) (23, 24), and ocular samples (vitreous, aqueous humor, corneal scrapings) (20, 21). Overall, Nanopore sequencing shows promising, consistent performance across sterile and non-sterile, liquid and solid matrices, from common (urine, blood) to rare (e.g., subgaleal fluid clinical samples).

Several case reports have demonstrated the clinical utility of nanopore sequencing in resolving challenging diagnostic scenarios, such as the diagnosis of fastidious, slow-growing, intracellular, or otherwise difficult-to-culture organisms. The clinical value of early identification in these cases is particularly high, as misdiagnosis or delayed diagnosis frequently leads to prolonged empiric treatment, worse outcomes, or increased biosafety risk to laboratory personnel handling hazardous cultures (25). These include the identification of *Tropheryma whipplei* in a patient initially misdiagnosed with chronic gastritis using targeted next-generation sequencing tNGS using nanopore (26), detection of *C. canimorsus* in a septic shock patient within 19 h compared to a conventional turnaround time of 6.25 days using whole-blood nanopore sequencing (27), and diagnosis of *Coxiella burnetii* infection in cases where all routine diagnostic tests were negative via tNGS (28). Additionally, shotgun nanopore sequencing enabled the identification of *Dibothriocephalus nihonkaiense* in an atypical geographic and clinical presentation (12). Importantly, in all of these cases, the sequencing results informed targeted antimicrobial or antiparasitic therapy, after which patients showed marked clinical improvement.

Similarly, in much more prevalent infections like tuberculosis (TB), ONT tNGS was used to detect species (*Mycobacterium tuberculosis* [MTB]) and antimicrobial resistance (AMR) profiles in <48 h (vs 4–6 weeks for culture) with up to 98% sensitivity (29). For instance, in a retrospective study of 172 patients (129 pulmonary TB [PTB], 43 non-TB) (30), ONT tNGS detected MTB in 112/129 PTB cases and correctly identified all 23 NTM infections, outperforming smear, culture, and Xpert MTB/RIF across diagnostic comparisons. Detection was highest in BALF (62.3%) and remained superior in smear-negative and early disease, while in pleural effusion samples from 13 tuberculous pleurisy cases, ONT tNGS detected MTB in 11 patients, substantially exceeding smear (0), culture (1), and Xpert MTB/RIF (2, 30). Though it is important to note that these results do not account for differences in pre-analytical steps, such as sample preparation, between the different approaches.

These findings are further supported by a recent meta-analysis of several clinical research studies demonstrating high diagnostic accuracy of ONT-based assays for MTB detection, with a pooled sensitivity of 88.61% and a specificity of 93.18%, alongside strong overall performance metrics, compared to both metagenomic sequencing and traditional methods (31). A comparable pattern emerges in bacterial pneumonia, where a separate meta-analysis reported a pooled sensitivity of 86.08% and a specificity of 84.97%, with accuracy improving further under specific conditions, such as ventilator-associated pneumonia, use of endotracheal aspirate samples, and larger study sizes (32). However, performance varied across platforms and applications in both conditions. For MTB, GridION demonstrated higher accuracy for species detection, while MinION was more widely used for drug resistance testing where sensitivity differed across drugs (e.g., higher for rifampicin and pyrazinamide, lower for isoniazid and ethambutol). On the contrary, for bacterial pneumonia, diagnostic accuracy varied by platform,with MinION associated with better performance and GridION with reduced accuracy. When metagenomic NGS (mNGS) was included as part of the reference standard, diagnostic accuracy was further reduced. Across both conditions, overall accuracy was further affected by study design, reference standards, and heterogeneity among included studies, with retrospective designs and certain reference standards consistently reducing performance estimates (31, 32).

Antimicrobial resistance (AMR) causes millions of infections and contributes to nearly 5 million deaths globally each year. Indeed, Nanopore sequencing-based mNGS has also enabled culture-independent AMR profiling across a range of clinically relevant organisms, including *Staphylococcus aureus* from orthopedic implant-associated infections (33) and *Pseudomonas aeruginosa*, where carbapenemase families (encoded by resistance genes, such as blaKPC, blaNDM, blaIMP, blaVIM, and blaOXA) are frequently co-located with additional resistance determinants conferring resistance to β-lactams, aminoglycosides, and quinolones (34). Here, Nanopore sequencing offers an added advantage of long reads that can resolve the plasmid structures through which resistance spreads most rapidly (35). However, standalone ONT mNGS-based AMR prediction currently lacks universally standardized interpretive criteria and regulatory approval for routine diagnostic use in most regions, though practice varies by setting. mNGS approaches are generally robust, as they support a range of bioinformatic classification strategies, including but not limited to k-mer-based methods (e.g., Kraken2 and Bracken), alignment-based approaches (e.g., Minimap2 with custom databases), and probabilistic methods (e.g., Kaiju), enabling accurate strain typing, haplotype reconstruction, and *de novo* assembly of repetitive elements (e.g., rRNA operons and insertion sequences) (36). However, this often also requires hybrid polishing with complementary short-read data or very high coverage (>100× ), which may not be feasible in low-biomass specimens (37). Alternatively, sequencing of culture isolates provides a more controlled context for comprehensive AMR profiling, particularly for complex resistance profiles requiring high sequencing depth (38–40). As such, when it comes to AMR determination, nanopore-based approaches are currently best positioned as complementary tools in complex or culture-negative cases, though they hold significant potential for reducing time-to-targeted therapy across diverse infectious disease settings.

In CNS infections, nanopore 16S sequencing identified the pathogen in 40 of 41 genuine bacterial meningitis cases, including 23 cases that were culture-negative (17). Similarly, in endophthalmitis and infectious uveitis, targeted nanopore sequencing achieved positivity rates of 89.3 and 100%, respectively, compared to 35.7 and 62.5% by conventional methods (41), with particular advantage in fungal detection. This pattern extends to lower respiratory tract infections, where metagenomic nanopore sequencing detected pathogens in up to 39.1% of culture-negative samples, including anaerobic species that failed to grow entirely (11). Across these settings, however, the inability to distinguish live from dead bacterial DNA, the difficulty of differentiating true pathogens from colonizers, and the influence of prior antibiotic exposure on results collectively underscore that nanopore sequencing is best positioned as a complementary tool, with clinical decisions remaining grounded in the broader diagnostic and clinical picture (11, 17). Nanopore sequencing workflows can also be affected by methodological biases at the sample extraction and library-preparation stages, which can influence the relative representation of organisms in metagenomic data and affect the detection of organisms, such as RNA viruses (42). In lower respiratory tract infections, targeted nanopore sequencing identified causative organisms missed by culture, including fastidious pathogens, such as *Chlamydia psittaci* and *Talaromyces marneffei* (43). Beyond bacterial infections, nanopore sequencing has broadened pathogen detection to include viruses and fungi, retrospectively detecting viruses in 46 sepsis patients where culture identified only one (44) and identifying various *Aspergillus species* and *Pneumocystis jirovecii* in respiratory samples where traditional (or we can also write culture/staining) methods performed poorly (45). WGS using MinION has also detected antiviral resistance mutations in Human Herpesvirus 1 (thymidine kinase and polymerase mutants) (46) and HIV (NNRTI, NRTI, and INSTI mutations) (47), emphasizing its adaptability across pathogen types. For fungal pathogens, tNGS can identify azole and echinocandin resistance markers in *Candida* spp. directly from positive blood cultures. This approach showed performance comparable to Sanger sequencing for key genes like ERG11 and FKS (48).

Microscopic detection-based diagnosis is a commonly used approach in parasite diagnostics, though the sensitivity of such assays is limited, especially at low parasite densities (49). For instance, when compared to PCR as a reference standard in endemic areas, the pooled sensitivity of microscopy for malaria detection has been estimated at approximately 53%, meaning that roughly half of PCR-confirmed infections may be missed by microscopy alone (49). Furthermore, morphology-based methods struggle with species differentiation, leading to misdiagnosis (e.g., *P. knowlesi* mistaken for *P. malariae*). To address these challenges, nanopore sequencing has been applied as a gap-filling diagnostic. For instance, using adaptive sampling, computationally enriching target DNA in real time by rejecting non-target reads, nanopore sequencing enabled direct whole-genome sequencing of *Plasmodium falciparum* from unenriched patient blood samples, achieving 2.7–5.8-fold enrichment of parasite DNA and covering >97% of the reference genome, even in samples with low parasitemia (0.09–0.60%) (50). Similarly, a LAMP-MinION amplicon sequencing assay targeting the 18S rRNA gene accurately discriminated all five human *Plasmodium* species across blood samples from malaria patients (49). Another study developed a targeted NGS test and successfully detected *Trypanosoma brucei rhodesiense*, *Plasmodium falciparum*, and *Babesia bovis* in human blood samples spiked with as few as one, four, and four parasites per microliter, respectively (51).

Overall, nanopore sequencing has demonstrated consistent clinical value as a mostly complementary, but sometimes sole, diagnostic tool, offering rapid, broad-spectrum pathogen detection across diverse infection types and specimen matrices where conventional methods frequently fall short. Continued standardization and prospective validation will be essential to fully realize its potential, particularly in resource-limited settings.

## APPLICATION OF NANOPORE SEQUENCING IN SURVEILLANCE AND ONE HEALTH

Nanopore sequencing has been explored for infectious disease surveillance across the One Health spectrum spanning human, animal, and environmental domains. One Health approach aims to prevent and address health challenges, such as emerging infectious diseases, antimicrobial resistance, and food safety threats (52). Applied at local to global levels, the One Health approach supports coordinated pathogen prevention, detection, and response efforts, helping to protect ecosystems and strengthen global health security through more equitable and sustainable solutions.

Globally, estimated economic losses due to infectious diseases in livestock exceed billions (53). Critically, an estimated 60–75% of emerging human infectious diseases originate from animal reservoirs (54). Rapidly changing environmental conditions, including habitat destruction, intensive livestock farming, and climate change, are shifting co-evolutionary dynamics in favor of zoonotic and vector-borne pathogens, increasing the risk of spillover events from wildlife to livestock to humans. The high population densities and low genetic diversity of modern livestock and crops make them particularly susceptible to emerging infectious diseases, while close human–animal contact accelerates the evolution of zoonotic disease (55). Nanopore sequencing has been applied to whole-genome characterization of high-priority zoonotic pathogens, including foot-and-mouth disease virus (FMDV) (56), African swine fever virus (ASFV) (57), monkeypox virus (58), MTB (59), and highly pathogenic avian influenza (60), as well as common zoonotic bacteria, such as *Campylobacter* sp. (61). Metagenomic ONT workflows have demonstrated higher sensitivity than broad-range and nested PCR for tick-borne virus detection in pooled ticks (62), have been used to survey gastroenteritis-associated bacteria in forest rodents (63), and have enabled identification of novel zoonotic viruses, such as two previously unknown hepatoviruses in shrews (64).

In environmental surveillance, mNGS has characterized AMR gene profiles in wastewater from industrial and agricultural sources (65, 66), discriminated human-adapted and avian influenza lineages in wastewater (67), and provided species-level pathogen tracking in river water using 16S rRNA sequencing (68). It has also been used to profile air microbiota in healthcare-adjacent built environments and detect pathogens in open-air market food products (69, 70).

Futher surveillance efforts via ONT include assessment of pathogen transmission dynamics and detection of “silent outbreaks” at early time points (71, 72). The portability and minimal infrastructure requirements of the MinION were particularly consequential in landmark deployments, including in-country sequencing during the 2014–2016 Ebola epidemic in West Africa (73) and metagenomic sequencing at the epicenter of the 2018 Lassa fever outbreak in Nigeria (73, 74). More broadly, whole-genome nanopore sequencing has been used for surveillance of high-consequence viral pathogens, including Nipah virus (75), SARS-CoV-2 variant tracking (76), and arboviruses, such as West Nile and Wesselsbron viruses (77, 78), while targeted nanopore sequencing approaches have been applied to human astrovirus (79), dengue virus (80), and monkeypox virus (mpox) (58). Importantly, this technology bypasses the need for time-consuming virus culture in a more cost-effective manner, which is particularly vital for highly pathogenic agents like Nipah virus that require BSL-4 facilities (75).

Similarly, studies have shown that nanopore sequencing can be used for rapid identification and typing of clonal bacterial outbreaks, such as *Salmonella* (81), methicillin-resistant *S. aureus* (MRSA) (72), and *K. variicola* (82), often achieving sufficient detail for infection prevention and control actions (72). Notably, a prospective nanopore-based surveillance in a neonatal ICU identified an MRSA outbreak within 13 days of the index case, enabling rapid containment (72), and WGS identified contaminated sink-traps as the source of a *K. michiganensis* neonatal outbreak (83).

While these studies highlight the value of genomic surveillance, the contribution of nanopore sequencing varies depending on the surrounding workflow and available infrastructure. In some settings, particularly those involving field deployment, limited laboratory capacity and space, contexts requiring accessible workflows, or pathogens requiring specialized containment facilities, the portability and culture-independent nature of nanopore sequencing directly enable surveillance activities that would otherwise be difficult to implement. In contrast, many studies performed in established laboratory environments primarily demonstrate the benefits of genomic epidemiology itself, and comparable results could potentially be achieved using alternative sequencing platforms given appropriate infrastructure and turnaround times.

## LIMITATIONS, ETHICS, AND SECURITY RAMIFICATIONS RELATED TO GENERATION OF NANOPORE SEQUENCING DATA, STORAGE, AND ANALYSIS WORKFLOWS

While highlighting anopore sequencing’s potential as a novel technology in laboratory tool and diagnostic platform, we also need to address its current limitations. ONT faces several technical and practical limitations that constrain routine clinical adoption. Although recent chemistries (e.g., R10.4.1 flow cells with Kit 14) and improved basecalling softwares have reduced raw read error rates to 0.5–2 and <0.5% for duplex reads, these remain higher than short-read Illumina sequencing approach (~0.1%) (7). In sample types that are rich in host DNA (e.g., blood and CSF), pathogen DNA can constitute less than 1% of total reads, which necessitates host depletion or target enrichment, adding to cost, complexity, and risk of sample loss (10, 37). These technical constraints are particularly relevant in One Health contexts, where sample types from wildlife, livestock, or environmental matrices often have low pathogen biomass, complex microbial backgrounds, and degraded nucleic acids (84). An additional limitation is that reference genomes for many animal and environmental species remain incomplete, complicating host depletion strategies and taxonomic assignment (84). New library preparation protocols now accommodate fragmented or low-input DNA, though optimal sequencing still favors high-molecular weight nucleic acids. Direct RNA sequencing remains hampered by 3′ bias, inefficient capture of non-polyadenylated or short RNAs, and lower throughput (85, 86). Critically, for routine diagnostics, ONT workflows are currently more expensive and labor-intensive than PCR assays. Additionally, despite promising validation studies, they lack standardized, regulatory-approved end-to-end pipelines for diagnostic use, limiting implementation in routine clinical laboratories (9). ONT sequencing, like all DNA-based methods, cannot distinguish viable from nonviable organisms, and genotype-phenotype discordance (e.g., silent mutations, uncharacterized regulators, or efflux-mediated resistance) limiting clinical interpretability (87).

The very features that make nanopore sequencing a pivotal tool for pathogen genomic surveillance, such as high portability, real-time analysis, and relative ease of use, also introduce important ethical, legal, and societal implications as sequencing moves out of regulated clinics and research institutions into decentralized and sometimes unregulated settings. This same accessibility and decentralized approach can, however, also lead to increased risks of unregulated and incorrect usage due to potential lack of standardized quality-control protocols. Despite these trade-offs, nanopore sequencing can be especially critical in lower-resource countries, particularly in Asia, where ONT platforms are commonly deployed to monitor high-priority threats, such as coronaviruses, TB, influenza viruses, AMR bacteria, and arboviruses (88). Moreover, beyond institutional settings, the accessibility and portability of nanopore sequencing hold promise for supporting genomic sovereignty, potentially enabling indigenous and at-risk communities to build localized sequencing capacity, generate population-relevant reference data, and conduct independent surveillance without reliance on external infrastructure. However, this remains largely aspirational, with significant barriers around training, data infrastructure, and sustained funding yet to be addressed.

From a cybersecurity standpoint, portable sequencers are inherently vulnerable because they rely on external host machines (e.g., laptops) for critical computational tasks like basecalling, broadening the attack surface beyond secure laboratory networks (88). This workflow exposes sensitive genomic data to threats that compromise confidentiality, integrity, and availability. Specific risks include unauthorized remote access, manipulation of basecalled sequences (leading to misleading or harmful diagnoses), and denial-of-service or ransomware attacks that could halt sequencing and result in the irreversible loss of irreplaceable clinical or outbreak samples (89).

Additionally, further challenges manifest acutely in low- and middle-income country surveillance efforts, where programs often struggle with limited sustainability due to heavy reliance on external funding (57% of total funds) (88), procurement barriers causing prolonged reagent resupply times (median 8 weeks), and significant gaps in in-country bioinformatics and data governance capacity. Addressing these financial, technical, and ethical constraints is essential to ensure that the potential benefits of nanopore sequencing for global public health are realized equitably, responsibly, and sustainably.

## CONCLUSION

Nanopore sequencing offers a promising and novel sequencing-based platform for innovation in diagnostics, with certain degrees of unique flexibility and scalability compared to other short-read technologies. It supports high throughput with a manageable TAT, making it suitable for clinical assay applications. The technology has shown strong potential for pathogen detection and genomic surveillance, with particularly high utility in diagnosis of fastidious organisms, outbreak response, and antimicrobial resistance profiling in clinical and epidemiological settings. Beyond these established applications, nanopore sequencing has broader relevance in One Health monitoring and surveillance. However, independent and self-sufficient clinical adoption is not yet routine. Evidence from current research, reports, and case studies suggests that improvements in patient outcomes are emerging, though often reported anecdotally (35). At the same time, technical challenges in low-biomass settings and metagenomic samples, as well as regulatory, bioinformatic, and equity-related gaps, remain important areas for continued development. In this context, nanopore sequencing is currently best positioned not as a standalone solution but as a valuable complementary tool in diagnostics and a powerful asset for surveillance. When thoughtfully integrated into existing workflows, it holds strong potential to transform infectious disease management.

## SUPPLEMENTAL MATERIAL

Table S1 jcm.00083-26-s0001.pdf Summary of studies evaluating nanopore sequencing for pathogen detection.

ASM does not own the copyrights to Supplemental Material that may be linked to, or accessed through, an article. The authors have granted ASM a non-exclusive, world-wide license to publish the Supplemental Material files. Please contact the corresponding author directly for reuse.
