# Supplementary material for: Nanopore sequencing in infectious disease diagnostics and surveillance
Source: J Clin Microbiol. 2026 Sep 17;64(10):e00083-26. doi: 10.1128/jcm.00083-26 (PMC13712941; doi:10.1128/jcm.00083-26)
Supplement: Supplementary file 1 [file jcm.00083-26-s0001.pdf]

Table S1. Summary of studies evaluating nanopore sequencing for pathogen detection.

Citation number	Paper title	Author/s (Year of Publication)	Clinical/Research study	Kind of study	Number of samples	Organisms detected	Disease	Type of samples	Sample extraction method	Confirmed application of ONT (device/model used)	Flow cell type	Nanopore sequencing type	Comparison to other diagnostics	Bioinformatic analysis done on	Limitations
10	Nanopore metagenomics enables rapid clinical diagnosis of bacterial lower respiratory infection	Charalampous, T., et al. (2019)	Clinical (Pilot and optimization); Applied field study	Diagnostics (Bacterial lower respiratory infections)	81 (n= 40 pilot, n= 41 optimized)	Metagenomic sample	Lower respiratory tract infection	Oropharyngeal swabs	Saponin-based host DNA depletion	Yes (MinION/ Model Unspecified)	R9.4, R9.5 or R9.4.1	Metagenomic sequencing	Culture (gold standard)	Centrifuge (taxonomic classification), CARD database (AMR gene detection), Minimap2, Canu (assembly), NanoOK, BRIG (genome comparison)	Small pilot size (81 samples)
11	Prospective Evaluation of a Rapid Clinical Metagenomics Test for Bacterial Pneumonia	Mu, S., et al. (2021)	Clinical (Prospective, single-center)	Diagnostics (Aetiological diagnosis evaluation)	292	Metagenomic sample	Lower respiratory tract infection	Bronchoalveolar lavage fluid and Sputum	DNA extraction with host depletion (processed on-site); library prep optimized with fastest available ONT kit (<1h prep)	Yes (MinION/model unspecified)	R9.5	Metagenomic sequencing	Routine culture, urinary antigen tests, qPCR, and Sanger sequencing	Species identification via Meta-ID criteria and contamination filtering	High cost (~$500/sample); 85.6% patients pre-treated with antibiotics; 34% samples negative by all methods
12	Molecular Identification of *Dibothriocephalus nihonkaiense* Infection Using Nanopore Sequencing: A Case Report and Literature Review	Tsang, H.F., et al.	Clinical (Applied field study)	Case report and literature review	Single patient sample	Dibothriocephalus nihonkaiense	*D. nihonkaiense* infection	*Ex vivo* parasite and egg recovered from patient	Qiagen TissueLyser III mechanical disruption (30Hz/15s) + QIAamp DNA Blood Mini Kit (Qiagen); 3x KAPA Pure bead cleanup; ONT Ligation Sequencing Kit SQK-LSK114	Yes (MinION Mk1C)	R9.4.1	Nanopore shotgun metagenomic sequencing	None (standalone ONT application), supplemented with routine X-ray and stool microscopy analysis	Dorado Basecalling (dna_r10.4.1_e8.2_400bps_sup@v4.3.0, 400 bases/s); CZID Nanopore mNGS Pipeline v0.7	Not specified
13	Rapid inference of antibiotic susceptibility phenotype of uropathogens using metagenomic sequencing with neighbor typing	Carroll, A. C., et al. (2025)	Research (Applied field study)	Metagenomic pathogen ID and antibiotic susceptibility profiling	279 (n= 131 (*Klebsiella spp*. including *Klebsiella pneumoniae, Klebsiella variicola, Klebsiella oxytoca, and Klebsiella aerogenes*) and n= 148 (*E. coli*) isolates)	*Klebsiella spp*. (including *Klebsiella pneumoniae, Klebsiella variicola, Klebsiella oxytoca, and Klebsiella aerogenes*), and *E. coli*	Urinary tract infection	Urine	Mechanical host-cell depletion, then DNA extraction and Qubit quantitation (specific kit not named in paper); libraries built with ONT Rapid PCR Barcoding Kit	Yes (MinION Mk1C)	R9.4.1	Metagenomic sequencing	None (standalone ONT application)	Kraken2 (v2.1.3) + KrakenTools, RefSeq DB (k2_standard_20230605), RASE pipeline (v0.1.0.0), Guppy (v6.4.6) onboard basecalling, R (v4.3.0) + ggplot2	Single geographic region; only two Enterobacterales genera; RASE cannot distinguish plasmid presence/absence; genotype-phenotype discordance possible
15	Identification of bacterial pathogens and antimicrobial resistance directly from clinical urines by nanopore-based metagenomic sequencing	Schmidt, K., et al.	Clinical (Applied field study)	Metagenomic pathogen ID and resistance profiling	15 (n= 10 clinical samples; n=5 healthy urine samples 'spiked' with MRD *E. coli*)	*Klebsiella pneumoniae, Enterobacter cloacae, E. coli*	Urinary tract infection	Urine	Bacterial Lysis Buffer (Roche), Proteinase K (14-22 mg/mL) (Roche), MagNA Pure Compact Nucleic Acid Isolation Kit (Roche), DNA Bacteria v3_2 protocol	Yes (MinION, model unspecified)		Metagenomic sequencing	Illumina HiSeq 2000	Poretools (HDF5-to-fasta extraction), BLAST search + CARD database (species/resistance gene ID), LAST aligner (CARD alignment, low-accuracy long-match params), in-house Perl scripts (taxa separation/taxonomy reporting)	Requires >10^7 CFU/mL input; one sample per flow cell; resistance point mutations not reliably detected; only single-pathogen UTIs tested
16	Optimizing DNA Extraction Methods for Nanopore Sequencing of *Neisseria gonorrhoeae* Directly from Urine Samples	Street, T. L., et al. (2020)	Clinical (retrospective), Applied field study	Novel (metagenomic sequencing-based) protocol and accuracy study	Unspecified	Neisseria gonorrhoeae	Urinary tract infection	Urine	Human cell/DNA depletion (centrifugation, saponin lysis, nuclease digestion, MolYsis Basic5 kit); compared against MagMAX, QIAamp UCP pathogen mini kit (best performer), and i-genomic urine kits	Yes (GridION)		Metagenomic sequencing	Conventional PCR	Guppy (v1.8.5/v3.3.0), Porechop (v0.2.4), Centrifuge (v1.0.4-beta), Minimap2 (v2.17), SAMtools (v1.9), CRuMPIT workflow, Python (pandas v0.25.1, seaborn v0.9.0, matplotlib v2.2.4)	Contamination risk from nonselective amplification; coverage thresholds for infection confirmation not established; larger validation studies needed
17	Nanopore 16S sequencing enhances the detection of bacterial meningitis after neurosurgery	Jang, Y., et al. (2022)	Clinical (Applied field study)	Nanopore microbial (16s) amplicon sequencing	285	*Klebsiella pneumoniae, Staphylococcus areus, Staphylococcus caprae*	Nosocomial bacterial meningisitis	Cerebrospinal fluid	DNA extracted using the PureLink Genomic DNA Mini Kit (Invitrogen). 16S rDNA PCR performed using Bacterial 16S rDNA PCR Kit (Takara)	Yes (MinION/model unspecified)	R9.5	Amplicon sequencing	Routine culture methods	Reads aligned to the BLAST database for taxonomic identification	Cannot distinguish live vs dead bacterial DNA; single-institution study; negative PCR determined by visual electrophoresis
18	Innovative approach of nanopore-based metagenomic sequencing for rapid identification of bacterial pathogens in joint fluid: A pilot study	Sung, Y. H., et al. (2024)	Clinical (Retrospective study)	Metagenomic sequencing, Point-of-care diagnostic assay design	11	*Cutibacterium acnes*	Bone and joint infection	Joint fluid	DNA extracted via zirconia/silica-bead beating, phenol-chloroform-isoamyl alcohol lysis, MagListo 5M Genomic DNA Extraction Kit (Bioneer)	Yes (MinION, model unspecified)	Unspecified	Amplicon sequencing	Routine culture methods	EPI2ME/Metrichor cloud platform (Metrichor Ltd., Oxford, UK)	Difficulty with polymicrobial infections; low culture-sequencing concordance; insufficient DNA input degrades performance
19	Molecular rapid diagnostic testing for bloodstream infections: Nanopore targeted sequencing with pathogen-specific primers	Han, D., et al. (2024)	Clinical study (Applied field study)	Assay optimization study	451	*Klebsiella pneumoniae, Human cytomegalovirus, Pseudomonas aeruginosa, Escherichia coli, Enterococcus faecium, Acinetobacter baumannii, Staphylococcus aureus*	Blood stream infection	Blood	Glass bead beating; automated extraction via Bloodstream Infection Pathogen Nucleic Acid Detection Kit (MD EasyDiagnosis); multiplex PCR (13 cycles) of pathogen-specific genes, magnetic bead purification, 2nd PCR (18 cycles) for indexing, ONT ligation/adapter kit, Sequencing Buffer II + Loading Beads II	Yes (GridION X5)	R9 Spot-ON	Targeted nanopore sequencing (tNGS)	Blood culture and antimicrobial susceptibility testing	Minimap2, IBM SPSS Statistics (SPSS 25)	Single-center validation; qualitative only; cannot distinguish ARG subtypes; no data on prior antibiotic exposure

20	Clinical performance of nanopore targeted sequencing for diagnosing endophthalmitis	Jing, D., et al. (2025)	Clinical study (Applied field study)	Field or clinical deployability testing	12	*S. epidermidis, Corynebacterium macginleyi, Malassezia restricta, Penicillium citrinum; E. faecalis; S. epidermidis, Rhodococcus qingshengii; S. epidermidis, S. capitis; S. pneumoniae, Streptococcus pseudopneumoniae; K. pneumoniae, Klebsiella variicola*	Endophthalmitis	Aqueous humor and/or vitreous samples	DNA extracted using Sansure DNA Extraction Kit (Changsha)	Yes (GridION X5)	Unspecified	Targeted nanopore sequencing (tNGS)	Conventional bacterial culture, Sanger sequencing	ont-guppy-for-gridion (v1.4.3-1, v3.0.3-1), BLAST, SPSS (23.0)	Small sample (n=12); single-center; short follow-up; contamination risk from targeted amplification
21	16S rRNA nanopore sequencing for the diagnosis of ocular infection: a feasibility study	Omi, M., et al. (2022)	Clinical (Applied field study)	Field or clinical deployability testing	6	*S. epidermidis, S. saccharolyticus, S. capitis, S. hominis, S. caprae, Moraxella nonliquefaciens, M. catarrhalis, M. lacunata, S. aureus, S. petrasii, Haemophilus influenzae, H. aegyptius, Cutibacterium acnes, Pseudomonas aeruginosa, Peptoniphilus lacydonensis, Ralstonia pickettii, Streptococcus pneumoniae, S. dysgalactiae, Finegoldia magna, S. mitis, Sphingomonas leidyi*	Corneal ulcer, endophthalmitis, conjunctival abscess	Corneal samples	DNA extracted using Maxwell RSC Blood DNA Kit (Promega, AS1400)	Yes (MinION Mk1B)	R9.4.1 Flongle cells	Nanopore microbial (16s) amplicon sequencing	Smear and culture method (additionally MALDI-TOF MS in one case)	Guppy (5.0.11), SeqKit (0.10.0), EPI2ME 16S workflow (2021.03.05), EPI2ME Desktop Agent (3.3.0)	Higher error rate vs MiSeq; multiple bacteria per sample risk false positives; no strain-level data
22	Rapid diagnosis of intra-amniotic infection using nanopore-based sequencing	Chaemsaithong, P., et al. (2023)	Clinical study (Applied field study)	Metagenomic pathogen ID; diagnostics (aetiological diagnosis evaluation)	10	*Streptococcus vaginalis, S. intermedius, S. constellatus; S. mitis, S. oralis, Peptoniphilus spp., Prevotella bivia, Ureaplasma urealyticum; Veillonella montpellierensis, Ureaplasma parvum, Veillonella atypica; Gardnerella vaginalis*	Intra-amniotic infection (Acute chorioamnionitis stage 2, grade 2)	Amniotic fluid	Not specified	Yes (MinION Mk1C)	R9.4.1	Nanopore microbial (16s) amplicon sequencing	16S rDNA PCR Sanger sequencing	Guppy (6.2.1, super accuracy model), Porechop (2.6.0), NanoFilt, EPI2ME	Not specified
23	16S rRNA long-read nanopore sequencing is feasible and reliable for endometrial microbiome analysis	Oberle, A., et al. (2021)	Cilinical (Prospective study)	Assay development and accuracy study (pilot study)	33	*Lactobacillus, Moraxella, Staphylococcus, Streptococcus, Kushneria, Cutibacterium, Burkholderia*	Endometrial health	Endometrial sample	PureLink Microbiome DNA Purification Kit (Thermo Fisher) used for ALL reported endometrial samples; DNeasy Blood & Tissue kit (Qiagen) was tested in an initial method-comparison step but was not the kit used downstream	Yes (MinION, model unspecified)	R9.4.1	Nanopore microbial (16s) amplicon sequencing	Conventional PCR and Illumina short-read Miniseq platform	Guppy, EPI2ME 16S workflow, R packages (microDecon, decontam)	30.3% samples uninterpretable; contaminant vs true taxon discrimination challenging; transcervical catheter carries contamination risk; small sample size
24	Genomic analysis of contaminant *Stenotrophomonas maltophilia* , from placental swab culture, carrying antibiotic resistance: a potential hospital laboratory contaminant	Pongchaikul, P., et al. (2025)	Clinical (Applied field study)	Field or clinical deployability testing	Single patient sample	*Stenotrophomonas maltophilia*	Acute chorioamnionitis	Vaginal swab, amniotic fluid obtained during Cesarean delivery, umbilical cord blood, and placental swab	Not specified	Yes (GridION)	R9.4.1	Nanopore microbial (16s) amplicon sequencing	Blood culture and antimicrobial susceptibility testing	Fastp (0.20.1), FastQC (0.11.8), Guppy (6.2.1, super accurate model), Porechop (0.2.4), NanoPlot (1.28.1), NanoFilt (2.5.0), Unicycler (0.4.8), Dfast, FastMLST, Mobile Element Finder, PHASTER, ResFinder (4.1), CRISPRCasFinder, VFanalyzer, Virulence Finder, PROKSEE	Sample could not be re-tested; environmental contamination source unconfirmed; AMR/biofilm gene causality unverified
25	Diagnosis of *Tropheryma whipplei* pneumonia using targeted nanopore sequencing: a rare case report and literature review	Yu, M., et al. (2025)	Clinical (Applied field study)	Targeted sequencing (tNGS) / Diagnosis	Single patient sample	*Tropheryma whipplei*	*Tropheryma whipplei* pneumonia	Bronchoalveolar lavage fluid	Not specified in abstract	Yes (Unspecified device/model)	Unspecified	Targeted nanopore sequencing (tNGS)	Bacterial culture (which was negative)	Not specified	Rarity of pathogen leads to common misdiagnosis
28	Diagnostic value of a nanopore sequencing assay of bronchoalveolar lavage fluid in pulmonary tuberculosis	Liu, Z., et al. (2023)	Clinical (Applied field study)	Diagnostics (Aetiological diagnosis evaluation)	55 (n= 29 Pulmonary TB, n= 26 non-PTB)	*Mycobacterium tuberculosis*	Pulmonary tuberculosis	Bronchoalveolar lavage fluid and sputum	gDNA extraction using established methods; quantified via Qubit 4.0 and Nanodrop	Yes (Unspecified device/model)	R9.4.1	Targeted sequencing of multiplex PCR amplicons (IS6110, rpoB, hsp65, gyrB)	MGIT culture and Xpert MTB/RIF	Guppy (basecalling); NanoPlot (QC); Medaka (variant calling); mapping to H37Rv	Small sample size; collection variables; host DNA noise; prior antibiotic use
29	Accuracy of Nanopore Sequencing as a Diagnostic Assay for Pulmonary Tuberculosis versus Smear, Culture and Xpert MTB/RIF: A Head-to-Head Comparison	Yang, J., et al. (2023)	Clinical (Applied field study)	Diagnostics (aetiological/ early diagnosis evaluation)	172 (n= 129 Pulmonary TB; n= 43 non-TB)	*Mycobacterium tuberculosis*	Pulmonary tuberculosis	Sputum (n= 8), Bronchoalveolar lavage fluid (n= 151), and pleural fluid (n= 13)	NaOH liquefaction (sputum), centrifugation, lysis solution, purification (Qubit/Nanodrop)	Yes (GridION)	R9.4.1	Nanopore sequencing (Multiplex amplicons targeting IS6110)	Smear, Culture, and Xpert MTB/RIF	Minimap2; human read removal (GRCh38); alignment to MTB reference (NC_000962.3)	Small sputum/pleural fluid subgroups; BALF collection variability; prior antibiotic use

43	Diagnostic value of nanopore-based metagenomic third-generation sequencing in the diagnosis of *Pneumocystis jirovecii* infection in patients with lung cancer	Luo, Y., et al. (2025)	Clinical (Prospective; observational study)	Diagnostic efficacy study	118	*Pneumocystis jirovecii*	Pneumocystis jirovecii Pneumonia (PJP, formerly known asd Pneumocystis carinii)	Bronchoalveolar lavage fluid	Not detailed in excerpt	Yes (MinION/ model unspecified)	R9.4, R9.5, R9.4.1	Metagenomic sequencing	Methenamine silver staining and real-time PCR	Pathogen identification via metagenomics	Interpretative complexity in mixed infections
47	A novel diagnostic method for malaria using loop-mediated isothermal amplification (LAMP) and MinION™ nanopore sequencer	Imai, K, et al. (2017)	Research (Applied field study)	Species identification scheme	549 (including n= 31 clinical)	*P. falciparum, P. vivax, P. ovale wallikeri, P. ovale curtisi, P. knowlesi and P. malariae*	Malaria	Clinical isolates and reference strains/genomes	Rapid Barcoding Sequencing Kit	Yes (MinION/model unspecified)	R9.4.1	Whole genome sequencing	Multilocus sequence analysis (MLSA)	WIMP (cloud-based) and Kraken2 pipeline with custom database	WIMP failed to identify 4 of 12 specific species
80	The rapid detection of a neonatal unit outbreak of a wild-type *Klebsiella variicola* using decentralized Oxford Nanopore sequencing	White, R.T., et al. (2025)	Research (Applied field study)	Outbreak investigation / Decentralized WGS,	4 (plus environmental)	*Klebsiella variicola*	Pneumonia (neonatal intensive care unit (NICU) outbreaks)	Bacteremia isolates and sink traps,	Subcultured isolates	Yes (MinION, model unspecified)	R9.4.1	Decentralized whole-genome sequencing (dWGS)	MDR surveillance (which failed to detect wild-type cases)	MLST and core-genome SNV phylogenetic analysis	Traditional methods misidentify K. variicola as K. pneumoniae; small sample (n=4)
